# Development of a Longitudinal Model for Disability Prediction in Older Adults in China: Analysis of CHARLS Data (2015-2020)

**DOI:** 10.2196/66723

**Published:** 2025-04-17

**Authors:** Jingjing Chu, Ying Li, Xinyi Wang, Qun Xu, Zherong Xu

**Affiliations:** 1 The First Affiliated Hospital, Zhejiang University School of Medicine, 79 Qingchun Road, Hangzhou, 310003, China, 86 057187236171; 2 Zhejiang University School of Medicine, Hangzhou, China

**Keywords:** disability, prediction model, older adults, China Health and Retirement Longitudinal Study, CHARLS, medical resources allocation

## Abstract

**Background:**

Disability profoundly affects older adults’ quality of life and imposes considerable burdens on health care systems in China’s aging society. Timely predictive models are essential for early intervention.

**Objective:**

We aimed to build effective predictive models of disability for early intervention and management in older adults in China, integrating physical, cognitive, physiological, and psychological factors.

**Methods:**

Data from the China Health and Retirement Longitudinal Study (CHARLS), spanning from 2015 to 2020 and involving 2450 older individuals initially in good health, were analyzed. The dataset was randomly divided into a training set with 70% data and a testing set with 30% data. LASSO regression with 10-fold cross-validation identified key predictors, which were then used to develop an Extreme Gradient Boosting (XGBoost) model. Model performance was evaluated using receiever operating characteristic curves, calibration curves, and clinical decision and impact curves. Variable contributions were interpreted using SHapley Additive exPlanations (SHAP) values.

**Results:**

LASSO regression was used to evaluate 36 potential predictors, resulting in a model incorporating 9 key variables: age, hand grip strength, standing balance, the 5-repetition chair stand test (CS-5), pain, depression, cognition, respiratory function, and comorbidities. The XGBoost model demonstrated an area under the curve of 0.846 (95% CI 0.825‐0.866) for the training set and 0.698 (95% CI 0.654‐0.743) for the testing set. Calibration curves demonstrated reliable predictive accuracy, with mean absolute errors of 0.001 and 0.011 for the training and testing sets, respectively. Clinical decision and impact curves demonstrated significant utility across risk thresholds. SHAP analysis identified pain, respiratory function, and age as top predictors, highlighting their substantial roles in disability risk. Hand grip and the CS-5 also significantly influenced the model. A web-based application was developed for personalized risk assessment and decision-making.

**Conclusion:**

A reliable predictive model for 5-year disability risk in Chinese older adults was developed and validated. This model enables the identification of high-risk individuals, supports early interventions, and optimizes resource allocation. Future efforts will focus on updating the model with new CHARLS data and validating it with external datasets.

## Introduction

The aging of the population presents a significant global challenge, with profound implications for health care systems, economic stability, and social services [1,2]. In China, where the older population is rapidly increasing, the prevalence of disability among older adults has become a pressing concern. According to the Chinese Centers for Disease Control and Prevention, the number of older individuals with disabilities reached 52.71 million in 2020 and is projected to exceed 77.65 million by 2030. By 2030, disabled older adults are expected to account for over 57% of the total disabled population, potentially rising to more than 70% by 2050 if no preventive measures are implemented [3]. Disability in older adults encompasses various limitations or difficulties in performing daily activities independently, typically due to chronic degenerative changes in function. It is commonly assessed through activities of daily living (ADL) and instrumental activities of daily living (IADL). This growing burden of disability impacts quality of life and places a significant strain on families and public resources. Therefore, accurate prediction of disability is crucial for early intervention and effective management.

Despite significant research efforts to forecast disability in older adults, existing models often lack sufficient precision and fail to account for the complex, multifactorial nature of disability. These models typically overlook the broader context of risk factors and offer limited utility for public health decision-making [4-6]. For instance, a study by Sun et al [5] identified depressive symptoms as a significant predictor across different types of disability. However, many existing models still fail to incorporate mental health factors alongside physical health indicators, limiting their real-world applicability. Furthermore, cognitive impairment has been found to be a strong predictor of disability in specific ADL and IADL tasks [6], highlighting the importance of integrating mental, cognitive, and physical health factors in predictive models. Disability, as a complex health issue, is influenced by multiple risk domains, including chronic diseases, polypharmacy, aging, mental health problems, unhealthy lifestyles, and the family social environment. A comprehensive predictive model should serve as a vital tool to improve early identification of at-risk individuals, inform public health strategies, and optimize resource allocation.

This study seeks to address the limitations in existing research by developing a disability prediction model specifically designed for the older Chinese population, using longitudinal data from the China Health and Retirement Longitudinal Study (CHARLS) collected between 2015 and 2020 [7]. While previous research has developed disability prediction models based on CHARLS data from 2015 to 2018 [8], the release of the 2020 survey data enables the extension of the analysis over a longer time frame. This study will leverage the validated Extreme Gradient Boosting (XGBoost) algorithm [8] to explore disability predictors in greater depth. In addition, by integrating variables such as sarcopenia and frailty-related indicators, which have previously been underexplored in predictive models for disability, we offer a more nuanced understanding of the physical, cognitive, physiological, and psychological factors contributing to disability risk. We aim to create a predictive model that not only offers high precision but also provides practical insights for health care professionals and policymakers.

## Methods

### Study Population

The data for this study were sourced from the CHARLS, initiated in 2011 by the National School of Development at Peking University. The CHARLS used a stratified, multistage Probability Proportional to Size random sampling method, covering 150 counties and 450 villages and urban communities across 28 provinces, involving 17,708 individuals from 10,257 households. Follow-up surveys were conducted in 2013, 2015, 2018, and 2020, with detailed methodology available in other publications [7].

Initially, 21,095 participants from the 2015 baseline survey were included. The final cohort consisted of 2450 individuals after applying the following exclusion criteria: (1) no information on biomarker or blood data; (2) younger than 60 years (for this study, older adults were defined as individuals aged 60 years or older, in accordance with the World Health Organization and the Chinese government’s standard for aging population classification); (3) missing ADL or IADL data in 2015; (4) missing follow-up ADL or IADL data in 2020; (5) having ADL and IADL limitations or any form of disability in 2015, including physical, intellectual, visual, auditory, or significant speech impairments; and (6) other relevant data missing.

### Ethical Considerations

The Institutional Review Board of Peking University (IRB No. IRB00001052-11014) approved the research, and all respondents provided informed consent. CHARLS adheres to the Declaration of Helsinki and China's Personal Information Protection Law. The CHARLS database adheres to strict privacy protection and anonymization principles during data collection and processing to ensure the security of participants' personal information.

### Assessment of Disability

Disability in this study was defined as impairment in performing ADL and IADL, which is commonly used in geriatric research to evaluate functional limitations in older adults. ADL assessed the ability to perform fundamental self-care tasks such as dressing, bathing, eating, getting out of bed, toileting, and managing urination and bowel movements. IADL measured more complex daily tasks, including household chores, cooking, shopping, phone use, financial management, and medication adherence. Responses were categorized into four levels: (1) no difficulty, (2) difficulty but can still do it, (3) difficulty and need help, and (4) cannot do it. To create a binary outcome, responses of (2) “Difficulty but can still do it,” (3) “Difficulty and need help,” and (4) “Cannot do it” were coded as 1 (indicating ADL and IADL disability), while the response “No difficulty” was coded as 0 (no disability). Participants were classified as having ADL and IADL disabilities if they reported any level of difficulty (levels 2‐4) in at least one ADL or IADL item [9].

### Predictive Variables

#### Clinical Factors

Laboratory assessments included a range of biomarkers: white blood cell count, hemoglobin, hematocrit, triglycerides, total cholesterol, glucose, uric acid, creatinine, blood urea nitrogen (BUN), high-density lipoprotein cholesterol, low-density lipoprotein cholesterol, cystatin C, C-reactive protein, and glycated hemoglobin.

Depressive symptoms were evaluated using the 10-item Center for Epidemiologic Studies Depression Scale (CES-D) [10]. CES-D scores ranged from 0 to 30, with higher scores indicating more severe symptoms. The CES-D has been validated for Chinese middle-aged and older populations [11]. A score of 10 or above was used to indicate depression, while scores below 10 indicated no depressive symptoms [12]. Cognitive function was assessed using a modified version of the telephone interview for cognitive status (TICS) questionnaire [13]. The overall cognitive score was calculated by summing the scores from four domains: (1) orientation (5 points), (2) computation (5 points), (3) memory (20 points), and (4) drawing (1 point), with a total possible score of 31 points [14]. Higher scores indicate better cognitive performance.

#### Physical Performance

The physical examination included measurements of systolic and diastolic blood pressure, pulse, and respiratory function. Respiratory function was measured using a peak flow meter. Participants were instructed to stand, take a deep breath, and blow as hard and fast as possible into the mouthpiece. The highest value from 3 attempts was recorded for analysis. Physical performance was assessed using gait speed, the 5-repetition chair stand test (CS-5), and standing balance. Gait speed was measured to evaluate lower limb function and mobility. A walking course of 2.5 meters was set up, and participants were instructed to walk the course twice at their usual pace. The average gait speed was calculated by dividing the distance by the time taken. The 5-repetition CS-5 was conducted to assess lower limb strength and endurance. Participants were asked to sit in a chair with their arms folded across their chest and, upon the examiner’s command, to stand up and sit down 5 times consecutively at their fastest pace without using their arms for support. The total time required to complete the 5 repetitions was recorded, with a longer duration indicating poorer lower limb function. The standing balance assessment involved maintaining a standing position for 10 seconds in three distinct foot placements: (1) side-by-side, (2) semitandem, and (3) full tandem. Handgrip strength, the primary indicator of muscle strength, was measured for each participant using a Yuejian WL-1000 dynamometer (Nantong Yuejian Physical Measurement Instrument Co). Handgrip strength was measured in both the dominant and nondominant hands, with 2 measurements per hand. The higher value for each hand was recorded, and the average value for the 2 hands was taken to represent the handgrip strength. Together, these assessments provided a comprehensive evaluation of physical function and performance.

Appendicular skeletal muscle mass (ASM) was estimated using a formula specifically developed for the Chinese population, which closely corresponds with dual-energy X-ray absorptiometry measurements [15,16]. The formula accounts for weight, height, sex (1 for males, 2 for females), and age as follows:

ASM =0.193× weight (kg) + 0.107 × height (cm) − 4.157 × sex − 0.037 × age − 2.631

### Potential Covariates

Covariates for our study were identified from previous literature and grouped into 2 main categories. The first included social and lifestyle factors: age, gender, BMI, marital status, residential area, daily sleep hours, and alcohol and tobacco use. BMI categories were defined as underweight (BMI <18.5 kg/m²), normal weight (BMI =18.5‐24 kg/m²), and overweight (BMI ≥24 kg/m²). The second category addressed pain, incidents of falling, and number of comorbidities (hypertension, dyslipidemia, diabetes, cancer, stroke, heart disease, lung disease, liver disease, kidney disease, digestive disease, mental health disorders, memory disorders, asthma, and arthritis) [14,17]. The comorbidity classification was based on the CHARLS questionnaire design. Neurological disorders, including Parkinson disease and Alzheimer disease, are included under memory-related diseases.

### Statistical Analysis

The preprocessed dataset was split into a training subset with 70% data and a testing subset with 30% data. Continuous variables were described using medians and IQR, with comparisons using the Mann–Whitney *U* test. Count variables were expressed as frequencies and percentages and assessed using the *χ*^2^ test. Model development and testing were performed using the training and testing sets, respectively.

Initial correlation analysis identified potential multicollinearity. Variable selection was conducted exclusively on the training set using LASSO regression with 10-fold cross-validation to prevent information leakage and ensure an unbiased evaluation of model performance. LASSO regression was chosen over other methods due to its ability to perform simultaneous variable selection and regularization, reduce overfitting, and enhance model interpretability. In addition, ablation experiments were conducted to evaluate the effect of removing specific features related to sarcopenia and frailty on model performance. The logloss metric was used to assess the performance of the models with and without these features. The selected variables informed the development of an XGBoost model, a machine learning algorithm that uses gradient boosting through decision trees to iteratively minimize prediction errors. For the optimization of the XGBoost model, hyperparameter tuning was performed using a grid search approach. Key hyperparameters were tuned, including the number of boosting rounds (nrounds), which determines the number of iterations for boosting, and the maximum tree depth, which controls the complexity of each individual tree. The learning rate (eta) was adjusted to control the weight of each update during training. In addition, the minimum loss reduction (gamma) for tree splitting, the feature subsampling ratio (colsample_bytree), and the minimum child weight were optimized to control the model’s complexity and prevent overfitting. The subsample ratio (subsample) was also tuned to control the fraction of training data used in each boosting round. The optimal parameters were selected based on the lowest logloss value obtained during cross-validation.

Model performance was assessed using receiver operating characteristic (ROC) curves and area under the curve (AUC) values, with higher AUC indicating better discrimination. Calibration curves evaluated the agreement between predicted and observed outcomes. Decision curve analysis (DCA) and clinical impact curves (CIC) aided in determining optimal application and estimating the model’s impact on patient management. SHapley Additive exPlanations (SHAP) values were used to interpret variable importance and model transparency. Four key SHAP plots were generated: (1) a summary plot, (2) dependence plot, (3) interaction plot, and (4) force plot. The model has been deployed on a web-based platform.

Analyses were conducted with R software (R Foundation for Statistical Computing), version 4.3.2. A *P* value <.05 was considered statistically significant.

## Results

### Baseline Characteristics

**Figure 1.**
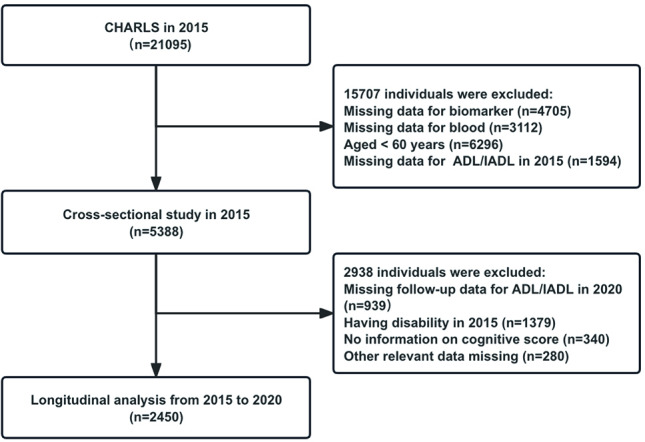
Flowchart of the data selection. CHARLS: China health and retirement longitudinal study, ADL/IADL: Activities of daily living and instrumental activities of daily living.

This study assessed a cohort of 2450 older adults initially in good health. Over a 5-year follow-up period, 610 participants developed disabilities, resulting in a disability incidence rate of 24.90%. The dataset was split 7:3, with the training set consisting of 1715 individuals (427 with disabilities) and the testing set comprising 735 participants (183 with disabilities). Baseline characteristics of both sets were detailed in Table 1 and Figure 1. Except for differences in sleep duration and white blood cell counts, no statistically significant differences were observed between the 2 groups (*P*>.05).

**Table 1.** Baseline characteristics in the training and testing subset.

Variable		Total	Training set	Testing set	*P* value
		N=2450	N=1715	N=735	
Dependent variable, n (%)					
Disability		610 (24.90)	427 (24.90)	183 (24.90)	—
Non-disability		1840 (75.10)	1288 (75.10)	552 (75.10)	—
ASM/Ht^2^		6.89 [5.97-7.59]	6.87 [5.96-7.56]	6.92 [6-7.70]	.12
Hand grip (kg)		31 [25-38]	31 [25-38]	31 [24.60-38]	.78
Gait speed (m/s)		0.83 [0.69-0.97]	0.83 [0.69-0.97]	0.83 [0.70-0.99]	.17
CS-5 (s)		8.78 [7.22-10.60]	8.81 [7.30-10.70]	8.71 [7.08-10.50]	.11
Balance		3 [3-3]	3 [3-3]	3 [3-3]	.99
Age (year)		65 [62-70]	65 [62-70]	65 [62-70]	.70
Sex, n (%)					.50
Male		1393 (56.86)	967 (56.38)	426 (57.96)	
Female		1057 (43.14)	748 (43.62)	309 (42.04)	
BMI level, n (%)					.14
Underweight		142 (5.80)	104 (6.06)	38 (5.17)	
Normal weight		1299 (53.02)	926 (54.00)	373 (50.75)	
Overweight		1009 (41.18)	685 (39.94)	324 (44.08)	
Marital status, n (%)					.21
Unmarried		417 (17.02)	303 (17.67)	114 (15.51)	
Married		2033 (82.98)	1412 (82.33)	621 (84.49)	
Living area, n (%)					.59
Rural		1958 (79.92)	1376 (80.23)	582 (79.18)	
Urban		492 (20.08)	339 (19.77)	153 (20.82)	
Education level, n (%)					.24
Illiterate		811 (33.10)	577 (33.64)	234 (31.84)	
Primary school		1062 (43.35)	753 (43.91)	309 (42.04)	
Middle school		388 (15.84)	256 (14.93)	132 (17.96)	
High school and above		189 (7.71)	129 (7.52)	60 (8.16)	
Sleeping, n (%)					.04^a^
<6 h		699 (28.53)	511 (29.80)	188 (25.58)	
≥6 h		1751 (71.47)	1204 (70.20)	547 (74.42)	
Pain, n (%):					.17
No		1981 (80.86)	1374 (80.12)	607 (82.59)	
Yes		469 (19.14)	341 (19.88)	128 (17.41)	
Falldown, n (%)					.81
No		2102 (85.80)	1469 (85.66)	633 (86.12)	
Yes		348 (14.20)	246 (14.34)	102 (13.88)	
Smoking, n (%)					.35
No		1497 (61.10)	1037 (60.47)	460 (62.59)	
Yes		953 (38.90)	678 (39.53)	275 (37.41)	
Drinking, n (%)					.45
No		1496 (61.06)	1056 (61.57)	440 (59.86)	
Yes		954 (38.94)	659 (38.43)	295 (40.14)	
Comorbidities, n (%)					.33
0		1705 (69.59)	1209 (70.50)	496 (67.48)	
1		538 (22.96)	365 (21.28)	173 (23.54)	
≥2		207 (8.45)	141 (8.22)	66 (8.98)	
Depression, n (%)					.37
No		1900 (77.55)	1339 (78.08)	561 (76.33)	
Yes		550 (22.45)	376 (21.92)	174 (23.67)	
Cognition		15.00 [12.00-18.00]	15.00 [11.50-18]	16 [12-19]	.46
Systolic BP^b^		129.67 [116.67-143.67]	129.67 [116.33-143.33]	130.33 [118-143.67]	.21
Diastolic BP		74.00 [67.33-81.33]	74 [67.33-81.33]	74.33 [67.33-81.83]	.71
Pulse		72.3 [66-79.3]	72.67 [66-79.67]	72 [66-79]	.53
Respiratory function		280 [203.33-360]	276.67[200-356.67]	280 [213-363.00]	.15
WBC^c^ (1000)		5.70 [4.75-6.80]	5.67 [4.72-6.78]	5.90 [4.80-6.90]	.01^a^
HGB^d^ (g/dl)		13.74 [12.66-14.80]	13.70 [12.60-14.80]	13.90 [12.70-14.90]	.13
HCT^e^ (%)		41.60 [38.50-45.00]	41.40 [38.40-44.80]	41.90 [38.70-45.20]	.19
TG^f^ (mg/dl)		110.62 [81.42-161.95]	109.73 [80.53-161.06]	114.16 [81.42-163.72]	.30
CHO^g^ (mg/dl)		182.63 [161-205.79]	183.40 [161.39-206.56]	181.47 [159.85-203.86]	.24
GLU^h^ (mg/dl)		97.30 [90.09-108.11]	95.50 [90.09-108.11]	97.30 [90.10-108.11]	.46
UA^i^ (mg/dl)		5.00 [4.10-5.90]	5.00 [4.10-5.90]	4.90 [4.10-5.90]	.51
CRP^j^ (mg/l)		1.40 [0.80-2.60]	1.40 [0.80-2.70]	1.40 [0.80-2.40]	.17
HbA1c^k^ (%)		5.90 [5.60-6.20]	5.90 [5.60-6.20]	5.90 [5.60-6.20]	.81
CREA^l^ (mg/dl)		0.81 [0.69-0.93]	0.80 [0.69-0.93]	0.81 [0.70-0.94]	.27
BUN^m^ (mg/dl)		15.13 [12.89-18.49]	15.41 [12.89-18.49]	15.13 [12.61-18.49]	.37
HDL^n^ (mg/dl)		50.19 [43.24-57.92]	50.19 [43.24-58.30]	50.19 [43.24-57.14]	.50
LDL^o^ (mg/dl)		101.93 [84.56-120.85]	102.32 [84.56-121.24]	99.61 [84.17-120.08]	.15

a *P*< .05

b BP: blood pressure.

c WBC: white blood cell.

d HGB: hemoglobin.

e HCT: hematocrit.

f TG: triglycerides.

g CHO: total cholesterol.

h GLU: glucose.

i UA: uric acid.

j CRP: C-reactive protein.

k HbA1c: glycated hemoglobin.

l CREA: creatinine.

m BUN: blood urea nitrogen.

n HDL: high-density lipoprotein cholesterol.

o LDL: low-density lipoprotein cholesterol.

### Predictor Selection

**Figure 2.**
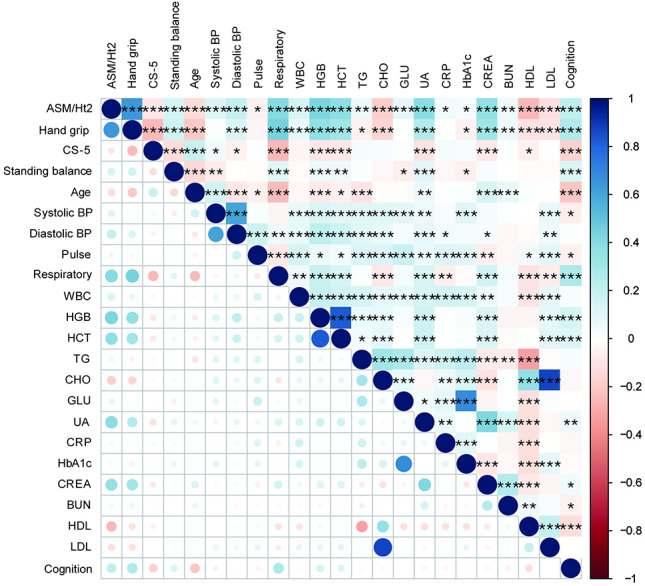
Correlation matrix of continuous independent variables. *: *P*<.05; **: *P*<.01; ***: *P*<.001. ASM/Ht2: appendicular skeletal muscle mass and height 2, CS-5: five-repetition chair stand test, Systolic and Diastolic BP: systolic blood pressure, WBC: white blood cell, HGB: hemoglobin, HCT: hematocrit, TG: triglycerides, CHO: total cholesterol, GLU: glucose, UA: uric acid, CRP: C-reactive protein, HbA1c: glycated hemoglobin, CREA: creatinine, BUN: blood urea nitrogen, HDL: high-density lipoprotein cholesterol, LDL: low-density lipoprotein cholesterol. Positive correlations are represented by blue tones, and negative correlations by red tones, with the intensity of the color indicating the strength of the correlation. Circle size is proportional to the absolute value of the correlation coefficient.

Figure 2 represented the interrelations among the continuous independent variables measured in the study. The matrix used varying shades of color and circle sizes to illustrate the magnitude and direction of correlation coefficients. The analysis revealed significant correlations, such as a negative association between hand grip and age, and a positive association between CHO and high-density lipoprotein (*P*<.001).

**Figure 3.**
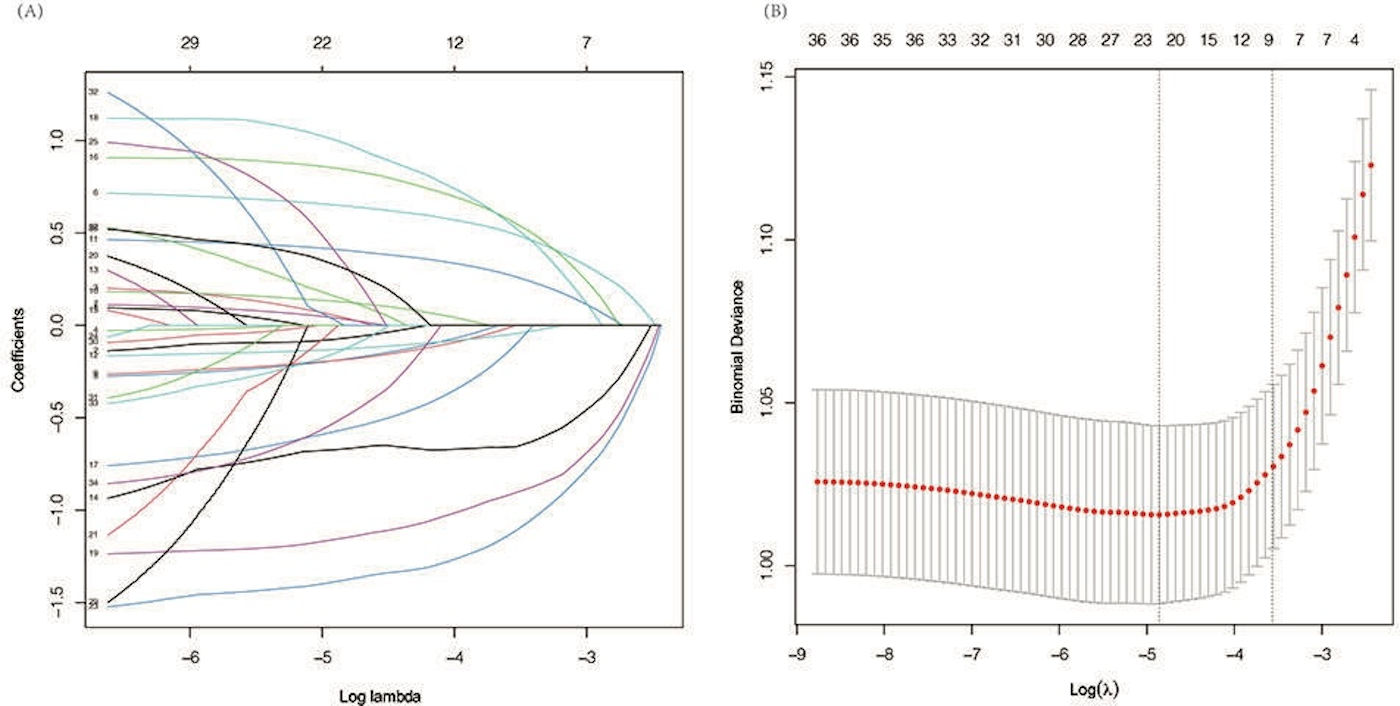
Variable selection via LASSO regression model. (A) Optimal parameter selection in LASSO regression. This plot illustrates the choice of the optimal λ, displaying log (λ) on the horizontal axis and regression coefficients on the vertical axis. (B) LASSO regression parameter (λ) selection via binomial deviance plot. Each point represents the model’s deviance at varying log (λ) values, with the vertical dotted line indicating the λ value that minimizes the binomial deviance.

To identify the strongest predictors of disabilities, the training dataset was normalized to account for different measurement units across variables. With disability as the dependent variable, 36 potential predictors were evaluated using LASSO regression. The compressive variable coefficient was used to avoid overfitting and improve predictive accuracy. The parameter λ was selected based on the largest λ within 1 SD of the minimal binomial deviance to enforce stricter penalty constraints. The LASSO regression retained 9 predictors with non-zero coefficients (Figure 3): age, hand grip, standing balance, CS-5, pain, depression, cognition, respiratory function, and the count of comorbidities.

Following LASSO regression, Table 2 summarizes the results of the ablation experiments, which evaluates the impact of removing specific sarcopenia- and frailty-related features (hand grip, CS-5, and standing balance) on model performance. Logloss was used as the primary metric to evaluate the model’s performance, with lower values indicating better predictive accuracy. The results showed that removing these features increased the logloss, with the most significant increase observed when all 3 features were removed simultaneously. These findings suggest that including sarcopenia-related parameters is crucial for maintaining the model’s predictive accuracy.

**Table 2.** Performance comparison of ablation experiments on model performance.

Model configuration	Logloss	Change	Number of features
All features (full model)	0.411	0	9
Removing hand grip	0.427	0.016	8
Removing CS-5^a^	0.421	0.010	8
Removing standing balance	0.413	0.002	8
Removing hand grip, CS-5, and standing balance	0.442	0.031	6

a CS-5: five-repetition chair stand test

### Construction and Assessment of the Predictive Model

Using disability outcomes from 2020 as the dependent variable and 9 predictors selected through LASSO regression, a predictive model was constructed using the XGBoost algorithm. The model’s hyperparameters were optimized through grid search and cross-validation. The best parameters identified were: nrounds=100, max_depth=3, eta=0.1, gamma=0.1, colsample_bytree=0.8, min_child_weight=3, and subsample=0.8. These parameters were used to train the final XGBoost model, which was then evaluated on the testing dataset.

**Figure 4.**
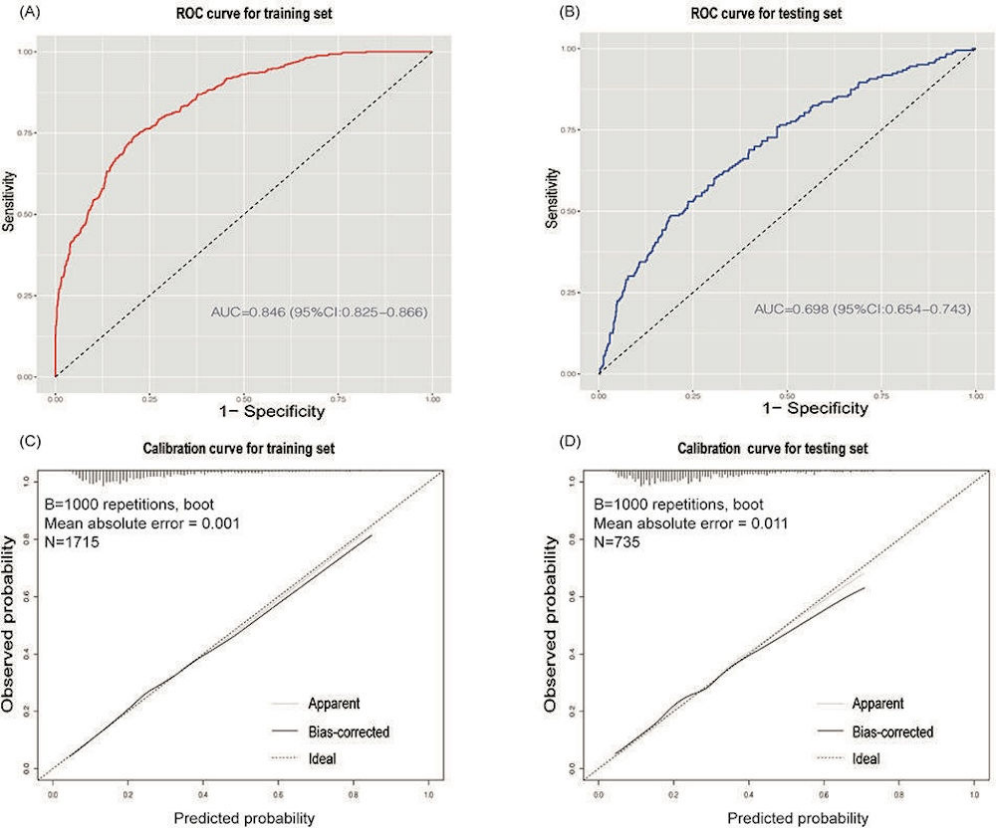
Receiver operating characteristic curves.

The performance of the XGBoost model was evaluated using ROC curves to assess its discrimination ability. In the training set, the model achieved an AUC of 0.846 (95% CI 0.825‐0.866), indicating good discrimination (Figure 4A). In the testing set, the AUC was 0.698 (95% CI 0.654‐0.743), reflecting moderate predictive accuracy (Figure 4B).

Calibration curves for the training and testing sets assessed the model’s predictive accuracy. The training set showed a mean absolute error of 0.001 (Figure 4C), suggesting high precision, while the testing set had a mean absolute error of 0.011 (Figure 4D). Both curves closely approximated the ideal line, confirming the model’s reliable prediction of disability risk.

**Figure 5.**
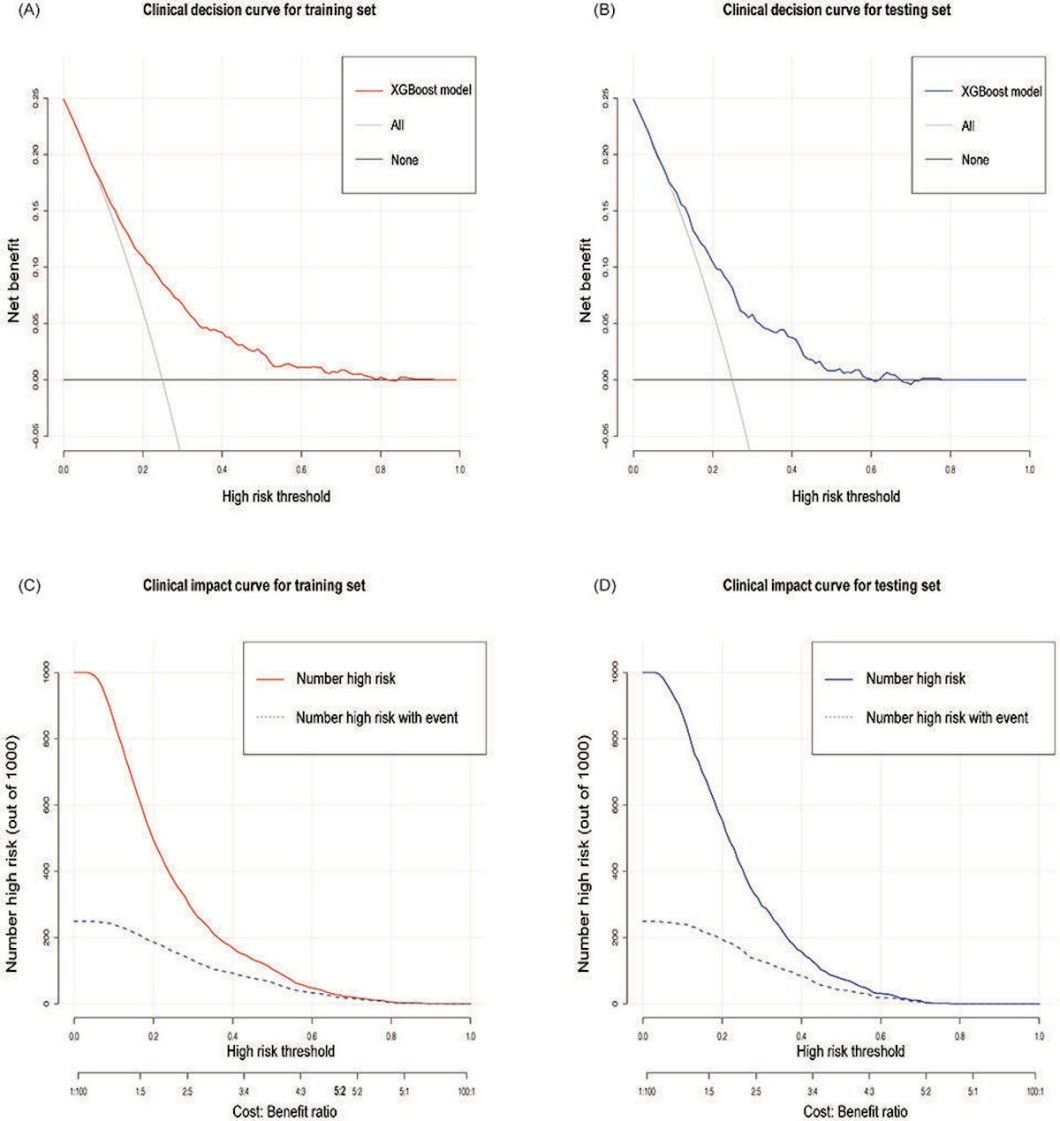
Clinical decision curves and impact curves for XGBoost model. (A) The red line represents the net benefit of the training set. (B) The blue line represents the net benefit of the testing set. The ’all’ line indicates the benefit when all patients are treated, and the ’none’ line when no patients are treated. (C-D) The solid lines depict the total number of individuals identified as high risk, and the dashed lines represent those at high risk who experienced the true event.

Clinical decision analysis demonstrated the effectiveness of the XGBoost model in predicting disability across different risk thresholds. In the training set, the DCA showed that using the XGBoost model to identify high-risk patients provided a net benefit (Figure 5A). For example, at a chosen risk threshold of 0.30, applying the model’s predictions would result in a better net benefit than treating all patients or treating none, highlighting the model’s clinical utility in improving decision-making. The testing set also demonstrated a similar net benefit (Figure 5B), confirming the model’s robustness and clinical applicability in an independent dataset.

In both datasets, the “Number high risk” line decreased steeply with increasing thresholds, indicating fewer individuals were classified as high-risk under stricter criteria (Figure 5C and D). In contrast, the “Number high risk with event” line, representing individuals who experienced disability, showed a more gradual decline. These trends highlight the model’s ability to focus predictions on a targeted group as thresholds increase, demonstrating its utility in guiding clinical decision-making and optimizing interventions for those most likely to benefit. For a more comprehensive evaluation of the model’s performance, the specificity, accuracy, positive predictive value (PPV), and negative predictive value at thresholds of 0.2 and 0.5 are provided in Multimedia Appendix 1.

### SHAP for Model Interpretation

**Figure 6.**
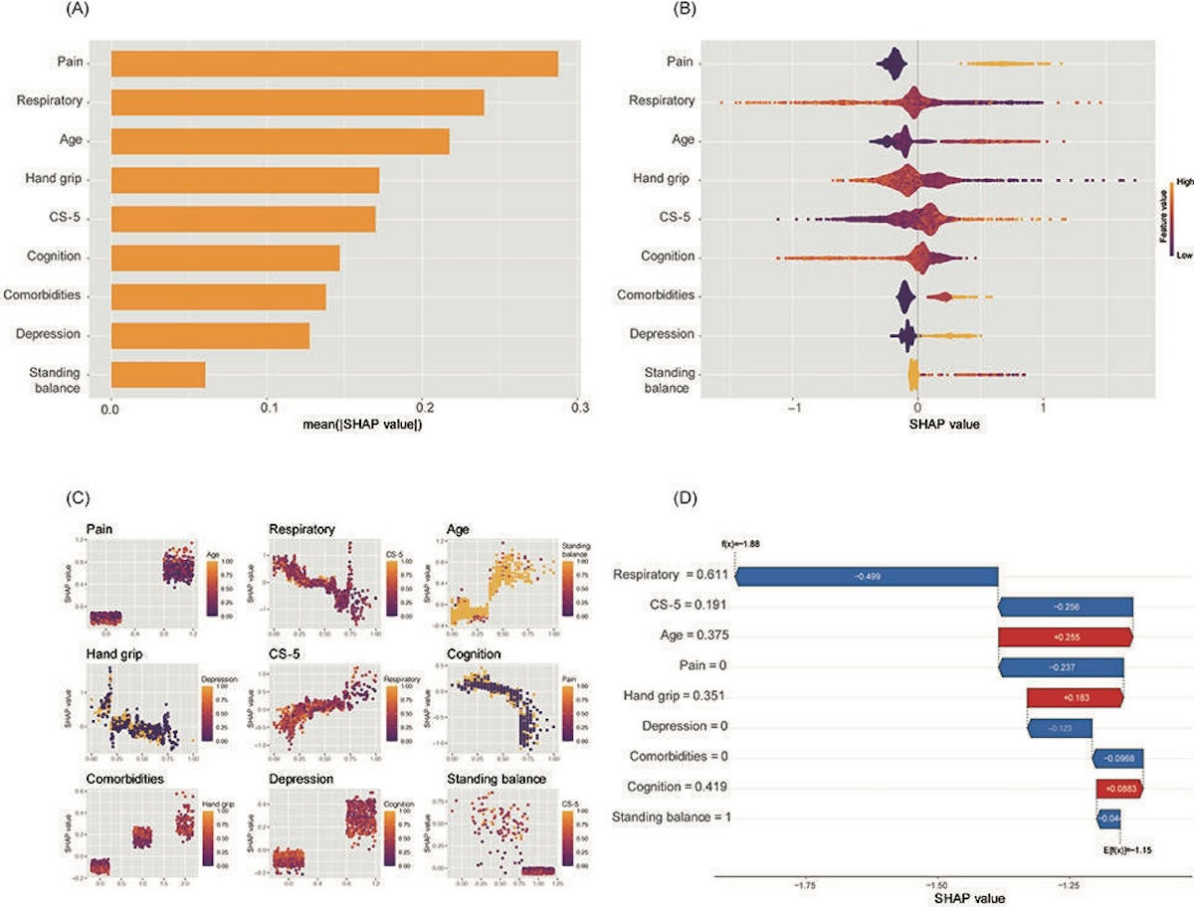
SHAP: SHapley additive exPlanations value interpretation diagram for predicting disability. (A) Variable importance in the predictive model as measured by SHAP values. (B) SHAP value distribution for predictive model variables. SHAP values for model variables are shown as violins. The color represents feature value intensity, and the width indicates impact density. (C) Scatter plot matrix of SHAP values for model predictors. Each plot reveals the influence of a single variable on the model output, with color intensity indicating the magnitude of the feature value. (D) Individual sample SHAP value analysis for disability risk prediction.

We used SHAP values to assess the influence of each variable on the 5-year disability risk. Figure 6A ranked predictors by their mean SHAP values, reflecting their average contribution to the model’s output. Pain had the highest mean SHAP value, followed by respiratory function and age, indicating their strong overall influence on disability prediction. Figure 6B showed the SHAP value distributions, where pain, respiratory function, and age exhibited the broadest ranges, suggesting their dynamic and individualized impact. Hand grip and CS-5 also significantly influenced the model, highlighting their importance in predicting physical function-related disability, while cognition, comorbidities, and depression showed more consistent contributions. Balance had the least impact.

The SHAP summary plot (Figure 6C) provided an overview of the overall influence of each predictor on the model’s output, revealing that pain, age, and respiratory function had the most substantial and wide-ranging influence on the predicted disability risk. The SHAP dependence plot (Figure 6D) visualized the individualized impact of these predictors on a single patient’s disability risk profile, offering insights into the model’s decision-making process at both macro and micro levels. Together, these plots provided a comprehensive understanding of the predictors’ contributions to disability risk prediction.

**Figure 7.**
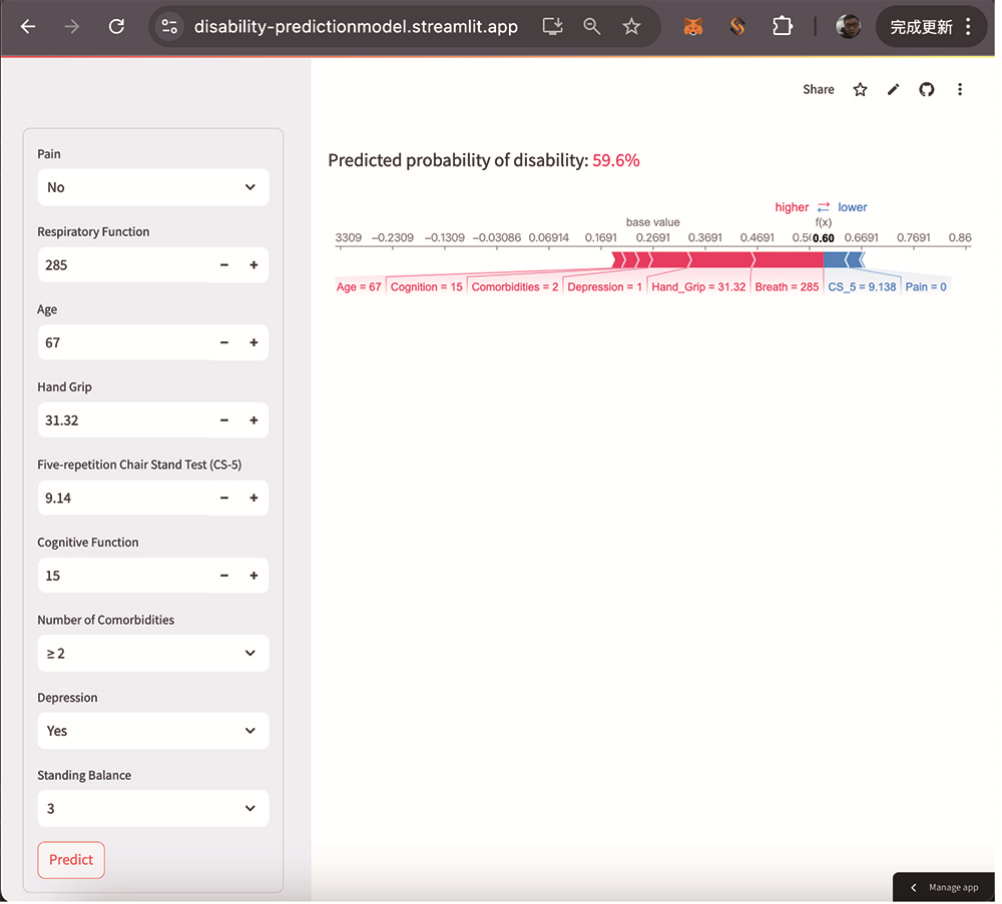
Online calculator for individual disability prediction.

A web-based calculator [18] enables clinicians to estimate the 5-year disability probability by entering patient-specific data [18], aiding in personalized clinical decisions (Figure 7).

## Discussion

### Principal Findings and Comparison With Previous Works

This study constructed an effective 5-year disability prediction model using baseline data from CHARLS 2015 to forecast disability occurrence in 2020. The model identified 9 key predictive variables (pain, respiratory function, age, handgrip strength, CS-5, cognitive function, depression, comorbidities, and standing balance) that are closely associated with disability incidence in Chinese older adults. Calibration curves demonstrated the model’s strong discrimination and consistency in both training and test sets, while DCA and CIC highlighted its positive clinical and social application value. The use of longitudinal data from CHARLS allowed for a more accurate, data-driven understanding of aging-related disability trends, leveraging demographic and health-related variables highly relevant to the Chinese context. In addition, the inclusion of sarcopenia and frailty-related diagnostic indicators as predictive variables represents a novel aspect of this research. These indicators enhance the model’s sensitivity to physical, cognitive, physiological, and psychological changes associated with aging that contribute to disability. This model offers a practical tool for improving disability prevention and management in older adults.

One notable observation in this study is the difference in AUC values between the training set (0.846) and the testing set (0.698). This discrepancy suggests potential overfitting, where the model may have captured noise or random fluctuations in the training data that do not generalize to unseen data. The observed discrepancy may be due to the imbalance in the dataset with respect to the outcome variable (disability status). To address this, we attempted oversampling to balance the data. However, this approach increased model complexity by retaining additional statistically significant predictors, raising concerns about overfitting. As a result, we proceeded with the original dataset to prioritize model simplicity and reduce overfitting risks. Although the model’s discriminative performance decreased in the test set, the overall trends remain robust, providing valuable insights into disability prediction for older populations. Moving forward, we plan to conduct external validation using additional datasets or longitudinal CHARLS follow-up data to further assess the model’s generalizability across diverse settings and time points. We selected the XGBoost algorithm for modeling based on its demonstrated ability to handle complex datasets and address class imbalance by assigning higher weights to the minority class [8]. This approach enhances the model’s ability to predict the minority class accurately. XGBoost’s advantages over other algorithms include its superior performance with structured and unstructured data, its regularization techniques to reduce overfitting, and its efficiency in training large datasets. In addition, its gradient boosting framework captures complex variable interactions, making it more suitable than traditional linear models. To optimize performance, we used advanced techniques such as cross-validation and hyperparameter tuning, including a comprehensive grid search over key parameters. This rigorous process ensured the model was robust and well-calibrated, improving its predictive performance. By extending the analysis to the latest 2020 CHARLS data, this study offers a more comprehensive 5-year prediction window compared with previous work focused on shorter time frames.

SHAP summary charts clarified the role and importance of each variable in predicting disability, providing transparency and interpretability to the model. According to the SHAP chart, pain, respiratory function, and age were the top 3 factors in importance, with the wide distribution of SHAP values. This indicates that changes in these variables significantly alter the risk of disability. Chronic pain, particularly lower back and neck pain, is a leading cause of disability globally, as highlighted by the Global Burden of Disease Study 2015. These types of pain are major causes of years lived with disability in many regions, including Latin America, the Caribbean, most regions of Asia, Oceania, and sub-Saharan Africa [19]. Chronic pain is closely linked to functional disability and poor physical performance in the older, as supported by various studies [20,21]. Respiratory function declines with age in older people, and respiratory impairment accounts for 20.7% of all types of disability [22]. Maximal inspiratory pressure and maximal expiratory pressure are correlated with hand-grip strength and skeletal muscle mass index [23,24]. Respiratory sarcopenia, characterized by a decrease in respiratory muscle strength alongside systemic skeletal muscle with aging [25], can lead to deterioration in respiratory force generation, adversely affecting activities of daily living [26]. Overall, respiratory impairment is prevalent among older individuals and is linked to physical inactivity and poor performance-based mobility [27]. Age is an independent risk factor for disability, with intrinsic capacity and functional ability declining with age. Disability levels are highest in the oldest patients [28], and age correlates with increased pain and respiratory impairment. Older adults are more likely to experience these issues, further increasing their disability risk [29]. In summary, higher pain scores, poorer respiratory function, and older age are associated with a greater risk of disability. Clinically, this suggests the need for emphasis on pain management and respiratory exercises in old people, particularly for those with chronic respiratory diseases.

The concentrated distribution of SHAP values for handgrip strength, CS-5, and cognitive function indicates these variables significantly influenced disability prediction. The ablation experiments further confirmed the impact of handgrip strength and CS-5 on the model. When these sarcopenia-related features were removed, the model’s performance was notably affected, as indicated by a significant increase in logloss. This finding aligns with the SHAP analysis results, which showed a wide distribution of values for these variables. Weak handgrip strength is identified as a key component of sarcopenia, strongly associated with subsequent disability and mortality [30]. Reduced handgrip strength and lower extremity strength, as measured by the CS-5, are strong predictors of functional impairment, disability, and low health-related quality of life, significantly increasing the risk of severe disability, frailty, and other health limitations in older adults [31,32]. Cognitive function is another crucial risk factor for disability. Studies have shown that cognitive decline is associated with ADL disability [33], and longitudinal research indicates that cognitive impairment may precede ADL disability, serving as a predictor of intermediate and late-stage ADL loss [34]. Physical and cognitive functions are closely related, with physical activity enhancing neurogenesis in the adult brain. Dual-task training, which enhances both cognitive and physical functions, has shown positive effects on cognitive function and physical activity in older individuals [35]. In summary, declines in handgrip strength, lower extremity strength (CS-5), and cognitive function are positively correlated with an increased risk of disability. This underscores the importance of targeted interventions, such as early muscle strength training for the upper and lower limbs and cognitive function exercises, to help reduce the risk of disability.

Although the roles of depression, comorbidities, and standing balance are less significant in predicting disability, they still contribute to its progression in the elderly and remain non-negligible factors. Depression, in particular, is a common psychological disorder among older adults and continues to be one of the most prevalent and disabling biopsychosocial conditions in this population. A Chinese cross-sectional study provided evidence of the association between depressive symptoms and ADL disability [36]. There is also a strong association between depression and physical activity, with significant mental health benefits gained from being physically active, even at levels below public health recommendations [37]. This may explain why depression can affect disability progression through physical function measures such as handgrip strength and CS-5. Similarly, comorbidities and standing balance are associated with disability and are critical factors in the multifactorial process of disability [38,39]. Comorbidity, the coexistence of 2 or more chronic diseases in older adults is a well-documented risk factor for increased mortality, reduced quality of life, and functional decline, ultimately leading to disability [40]. As a consequence of managing multiple chronic conditions, polypharmacy, defined as the concurrent use of multiple medications, becomes increasingly common in older populations [41]. Polypharmacy has been associated with a higher risk of falls, frailty, cognitive impairment, and adverse drug interactions, further exacerbating health deterioration and disability [42]. However, in this study, medication use was categorized in the CHARLS questionnaire only as Chinese traditional medicine or Western modern medicine, without detailed data on specific medications. This limitation prevented a comprehensive analysis of polypharmacy’s impact. The decline in standing balance in older adults indicates decreased postural control and increased risk of falls, often seen in populations with sarcopenia and frailty, which eventually progresses to disability [43]. Overall, these findings suggest that we should consider the combined effects of mental health, management of multiple chronic diseases, and balance function when predicting disability and formulating prevention strategies.

In our study, we evaluated the treatment benefits of the model using DCA. Figure 6A shows the net benefit across different risk thresholds. At low thresholds (<0.2), the net benefit was high but gradually declined as the threshold increased, approaching zero around 0.6. The model outperformed both all-treatment and no-treatment strategies across most thresholds, demonstrating its clinical utility. In practice, selecting an appropriate threshold is critical for clinical decision-making and resource allocation. A low threshold is suitable for high-sensitivity scenarios, such as community screening for early intervention in older adults. A medium threshold balances sensitivity and specificity, making it ideal for resource-limited settings where the model can precisely identify high-risk individuals for targeted interventions. Many clinicians have used DCA to test various disease prediction models, such as those for 30-day mortality in MIMIC-III patients with sepsis-3, major adverse cardiovascular events in older patients, and hypertension risk in patients with type 2 diabetes mellitus [44-46].

We evaluated the model’s predictive efficacy at different risk thresholds using CIC. As shown in Figure 6C, at thresholds below 0.2, the model identifies over 500 high-risk patients, with approximately 200 actual events, resulting in a high false-positive rate and increased resource consumption. This range is suitable for early widespread screening when follow-up resources are available. At thresholds between 0.2 and 0.5, the number of high-risk patients identified aligns more closely with actual events, balancing sensitivity and specificity while improving cost-effectiveness. This range is ideal for resource-limited settings. At thresholds above 0.5, the number of high-risk patients decreases significantly, nearly matching actual events but potentially missing some high-risk cases. The CIC provides clinicians with a visual tool to balance sensitivity and specificity, optimizing disability prediction and intervention strategies in older adults. CIC is commonly used to evaluate the predictive accuracy and clinical value of clinical prediction model for various diseases [47-49]. However, they are rarely used to evaluate the clinical usefulness of disability prediction models in older adults.

The 9 variables selected through LASSO regression form a streamlined yet effective set of predictors that can be easily integrated into routine clinical practice. Specifically, the inclusion of sarcopenia and frailty-related features provides health care professionals with clear and actionable insights into the ability of older adults to live independently, enabling timely interventions to prevent disability. By focusing on these key variables, the model remains interpretable, reducing the risk of “black-box” complexity in clinical decision-making. To facilitate practical application, we developed a web-based application via the Streamlit platform that uses these 9 predictors to calculate the 5-year risk of disability for individual patients. This user-friendly tool allows clinicians to input patient-specific data and receive immediate risk assessments, integrating predictive analytics into clinical workflows and bridging complex data models with everyday decision-making. Future applications of this model can aid healthcare professionals in identifying individuals at high risk of disability and implementing early, targeted interventions. This approach has the potential to delay the onset of disability and improve the quality of life for older individuals.

### Limitations

The limitations of this study include the selection of predictor variables. While the selected predictors are based on the best available evidence, other important variables, such as activity intensity, were not included due to high missing values. In addition, the model generalization and optimization is a limitation. The model performs well on the internal test set but lacks external validation due to the unavailability of a suitable external dataset. We plan to collect data from multicenter older care communities for external validation to further improve and optimize the model. As the CHARLS database updates, the model may need periodic updates to maintain accuracy and usefulness. Moreover, predicting long-term disability risk is challenging due to complex time interactions that may alter the risk trajectory.

### Conclusions

Our research incorporates parameters aligned with the diagnostic criteria for sarcopenia and frailty. These physical function measures are combined with predictors from cognitive and psychological health dimensions, recognizing the complex interplay of physical capability, aging, and mental health in the development of disability. This approach enhances the model’s precision and considers the need for the efficient identification of at-risk individuals and the optimization of medical resources in clinical practice. Consequently, the model provides a highly reliable disability prediction tool for older patients, health care workers, and policymakers. In the future, we will adjust the model based on updates to the CHARLS database to ensure its suitability for the older population in China. In addition, we will seek appropriate external databases for validation and promote the model’s application across different ethnic groups.

## Supplementary material

10.2196/66723 Multimedia Appendix 1 Model performance metrics at thresholds of 0.2 and 0.5 for the training and testing sets, respectively.

## Abbreviations

ADL: activities of daily living
ASM: appendicular skeletal muscle mass
AUC: area under the curve
CES-D: Center for Epidemiologic Studies Depression Scale
CHARLS: China Health and Retirement Longitudinal Study
CIC: clinical impact curve
CS-5: chair stand test
DCA: decision curve analysis
IADL: instrumental activities of daily living
LDL: low-density lipoprotein
ROC: receiver operating characteristic
SHAP: SHapley Additive exPlanations
TICS: telephone interview for cognitive status
