# Supplementary material for: Development of a Longitudinal Model for Disability Prediction in Older Adults in China: Analysis of CHARLS Data (2015-2020)
Source: JMIR Aging. 2025 Apr 17;8:e66723. doi: 10.2196/66723 (PMC12021300; doi:10.2196/66723)
Supplement: Multimedia Appendix 1 [file aging-v8-e66723-s001.doc]

Supplementary Table 1 Model Performance Metrics at Thresholds of 0.2 and 0.5 for the Training and Testing Sets

| Metrics | Training Set | | Testing Set | |
| --- | --- | --- | --- | --- |
|  | Threshold 0.2 | Threshold.0.5 | Threshold 0.2 | Threshold 0.5 |
| Specificity | 0.646 | 0.967 | 0.580 | 0.944 |
| Accuracy | 0.694 | 0.815 | 0.607 | 0.759 |
| PPV | 0.440 | 0.782 | 0.352 | 0.744 |
| NPV | 0.924 | 0.819 | 0.849 | 0.781 |

PPV: Positive Predictive Value；

NPV: Negative Predictive Value
